# Spiritual Care Empowerment and Midwives’ Adherence to the Maternal Rights Charter: A Quasiexperimental Study Using 360‐Degree Evaluation

**DOI:** 10.1155/jonm/2125073

**Published:** 2026-07-24

**Authors:** Soheila Jafarzadeh, Zahra Alipour, Hoda Ahmari Tehran, Zohre Khalajinia

**Affiliations:** ^1^ Department of Midwifery, School of Medicine, Qom University of Medical Sciences, Qom, Iran, muq.ac.ir; ^2^ Spiritual Health Research Center, Qom University of Medical Sciences, Qom, Iran, muq.ac.ir

**Keywords:** charter, empowerment, medicine, peer support, professional development, spiritual care

## Abstract

**Aim:**

This study examined whether participation in a spiritual care empowerment programme was associated with changes in midwives’ adherence to the Maternal Rights Charter using a 360‐degree evaluation.

**Background:**

Adherence to maternal rights reflects respectful, person‐centred, dignity‐preserving maternity care. Spiritual care empowerment may enhance relational and communication competencies, but its association with observable adherence to the Maternal Rights Charter remains underexplored.

**Methods:**

This quasiexperimental, pretest–posttest control group study was conducted in a labour and delivery ward in Qom, Iran, from May to August 2023. Fifty‐six eligible midwives were recruited through convenience sampling and assigned to the intervention or control group using simple random allocation by lottery. The final complete‐case analysis included 51 midwives: 23 intervention and 28 control. The intervention group participated in an 8‐h spiritual care empowerment workshop over 2 days, followed by 2 weeks of educational support; the control group received routine education only. Adherence was evaluated at baseline, 2 weeks and 2 months postworkshop using self, peer, supervisor and mother assessments. Data were analysed using two‐way repeated‐measures analysis of variance.

**Results:**

Significant time‐by‐group interactions were observed for total scores across all evaluator perspectives: self‐assessment (*F* = 22.38, *p* < 0.001, *η* *p* ^2^ = 0.314), peer assessment (*F* = 29.72, *p* < 0.001, *η* *p* ^2^ = 0.378), supervisor assessment (*F* = 41.74, *p* < 0.001, *η* *p* ^2^ = 0.460) and mother assessment (*F* = 21.56, *p* < 0.001, *η* *p* ^2^ = 0.306).

**Conclusion:**

Participation in the spiritual care empowerment programme was associated with improved midwives’ adherence scores for the Maternal Rights Charter across evaluator perspectives.

**Implications for Nursing Management:**

Nursing and midwifery managers may consider structured spiritual care empowerment as one component of continuing professional development. Multisource assessment, including mothers’ perspectives, may help identify training needs and monitor changes in rights‐based maternity care.

## 1. Introduction

Respecting women’s rights during childbirth is a core element of respectful, person‐centred maternity care. These approaches define high‐quality childbirth care through dignity, privacy, confidentiality, informed consent, communication, autonomy, safety and freedom from mistreatment [1–3]. For nursing and midwifery management in Iran, adherence to the Maternal Rights Charter is more than an ethical principle; it is an observable aspect of maternity care quality reflected in respectful communication, information provision, privacy protection, professional behaviour and women’s perception of respectful maternity care [2, 4, 5].

Despite the growing attention to respectful maternity care, mistreatment and disrespect during childbirth remain important concerns. A global synthesis reported that they affect a substantial proportion of women [6]. Evidence from Iran has highlighted the importance of information provision, respectful staff behaviour and women’s participation in childbirth care and decision‐making [4, 7, 8]. In a recent qualitative study of health workers who had undergone elective caesarean sections, the preference for elective caesarean sections was shaped by safety concerns and the anticipated loss of privacy and dignity [7].

The theoretical framework of this study integrates respectful maternity care, person‐centred maternity care, health system responsiveness and spiritual care competence [1, 3, 9, 10]. These perspectives share a focus on dignity, informed choice, privacy and confidentiality, communication, timely care and support as dimensions of the quality of maternal healthcare [2, 3, 9]. In the Iranian context, qualitative evidence on professional ethics has highlighted the importance of professional values, patients’ rights, respectful communication, accountability and teamwork in clinical practice [5], while research on health system reform points to broader structural and organisational conditions that may influence service delivery [11].

In the present study, adherence to the Maternal Rights Charter was operationalised through midwives’ communication, respect for privacy, information provision, respectful presence and responses to women’s emotional and physical needs [2, 3, 12]. Therefore, educational interventions designed to support adherence to maternal rights should strengthen relational competencies through which these rights become visible in clinical encounters, not only awareness of rights [5, 10, 13, 14].

Spiritual care competence may provide a relevant educational pathway for strengthening these skills. Spiritual care in nursing addresses meaning, values, beliefs, dignity, emotional security, cultural sensitivity and recognition of the patient as a whole [10, 15]. Consistent with this view, Khalajinia et al. [16] reported that healthcare providers in Iran perceived spiritual care as closely linked to observing patients’ rights, including respect, the right to choose, privacy preservation, proper communication, professionalism and attention to emotional and psychological needs. These concepts overlap with rights‐based maternity behaviours, including respectful communication, confidentiality, compassionate presence, emotional support and sensitivity to women’s values and beliefs [1, 2, 10]. Accordingly, spiritual care empowerment may support adherence to the Maternal Rights Charter by strengthening relational attentiveness, respectful communication, spiritual assessment and responsiveness to women’s emotional, informational, privacy‐related and dignity‐related needs [2, 10, 15]. Evidence shows that nurses’ perceptions of spirituality and spiritual care and their spiritual care competencies are generally moderate; education and training are important correlates, while some studies also report associations with clinical experience and speciality area [10, 17].

Provider training is frequently used to promote respectful maternity care, but programmes vary in content, delivery, evaluation and feedback mechanisms [13, 14, 18]. Many available evaluations use heterogeneous tools and often rely on limited evaluator perspectives, which may not fully reflect observable professional behaviour in ethically sensitive clinical settings [1, 2]. Multisource or 360‐degree evaluations can provide broader feedback on workplace performance by incorporating more than one perspective [19]. In the Iranian maternity context, Safaee et al. [12] used a 360‐degree evaluation approach to assess respect for the Bill of Mother’s Rights in labour and delivery by midwives responsible for delivery, supporting the relevance of this assessment framework for evaluating maternal rights observance in maternity settings.

Limited evidence has examined whether empowering midwives in spiritual care is associated with improved adherence to the Maternal Rights Charter when adherence is assessed using a 360‐degree framework. Accordingly, this quasiexperimental study aimed to examine whether participation in a spiritual care empowerment programme was associated with changes in midwives’ adherence to the Maternal Rights Charter in a maternity setting in Iran, using a 360‐degree evaluation approach previously applied to the assessment of maternal rights observance in Iranian labour and delivery settings and focussing on emotional, physical and informational/counselling dimensions of maternal rights observance over time [12].

## 2. Methods

### 2.1. Study Design and Setting

This quasiexperimental study used a pretest–posttest control group design. The study was conducted in the labour and delivery ward of Khayerin Salamat Educational and Medical Centre, Qom, Iran, from May to August 2023. The reporting of this study followed the Transparent Reporting of Evaluations with Nonrandomised Designs (TREND) statement [20]. The completed TREND checklist is provided as Supporting File 1.

### 2.2. Participants

The study population comprised midwives working in the labour and delivery ward and mothers under their care. Midwives were recruited using convenience sampling, and all eligible midwives who agreed to participate were consecutively enrolled. The inclusion criteria were holding at least a bachelor’s degree in midwifery, working in the delivery room across morning, evening or night shifts and having at least 1 year of delivery room experience. The exclusion criteria were nonattendance at the empowerment programme, absence from the workshop for more than 2 h, incomplete questionnaires or inability to complete follow‐up assessments.

**FIGURE 1:**
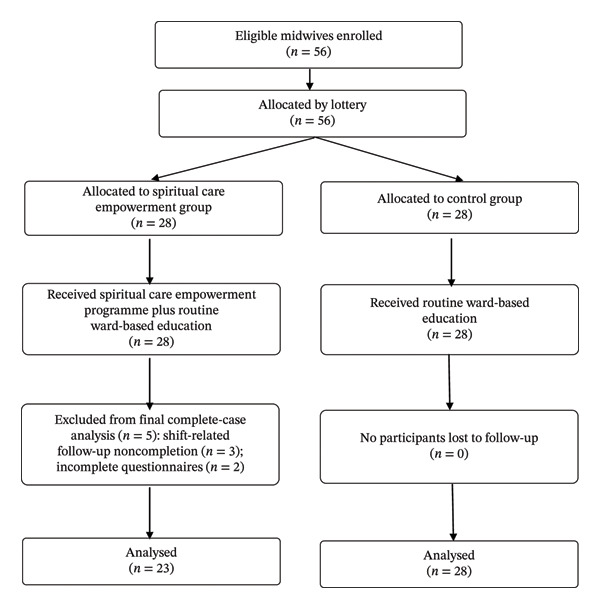
Recruitment, allocation, follow‐up and complete‐case analysis of participating midwives.

After enrolment, the participants were assigned to the intervention or control group in a 1:1 ratio using simple random allocation by lottery. A total of 56 midwives were enrolled, with 28 initially allocated to each group. During follow‐up, five midwives from the intervention group were excluded from the final analysis: three because their work shifts could not be coordinated with the scheduled follow‐up assessments and two because of incomplete questionnaires. Of the three shift‐related exclusions, two occurred at the 2‐week follow‐up and one at the 2‐month follow‐up period. No participants were lost in the control group. The final complete‐case sample included 23 midwives in the intervention group and 28 in the control group. A flow diagram of recruitment, allocation, follow‐up and complete‐case analysis is presented in Figure 1.

### 2.3. Sample Size

The sample size was determined a priori using previous Iranian quasiexperimental evidence on patient rights education in maternity care. Bayrami et al. [21] reported postintervention physical patient rights scores of 94.7 ± 6.54 in the intervention group and 82.5 ± 10.4 in the control group. Given the indirect applicability of that study to the present research, including differences in the study population, outcome instrument, 360‐degree evaluation framework and repeated‐measures design, the estimate was applied conservatively. A minimum of 22 midwives per group was targeted, and 28 midwives were recruited into each group at baseline to compensate for potential attrition.

### 2.4. Instruments

Data were collected using two instruments: (1) a demographic questionnaire assessing age, marital status, employment type, organisational position, income, work experience, working hours and education level; and (2) the Observance of the Maternal Rights Charter in Labour Questionnaire.

Observance of the Maternal Rights Charter in Labour Questionnaire: This 34‐item questionnaire was adopted from the instrument reported by Safaee et al. [12], which was developed based on the Iranian Ministry of Health’s Charter of Mothers’ Rights in Labour and Delivery. The questionnaire assesses three dimensions: emotional support (14 items), physical support (9 items) and informational/counselling support (11 items). In the original report, the instrument content was reviewed for content validity, and its stability was assessed using the test–retest method. The content validity of the tool was confirmed by Safaee et al. [12], and its reliability was established using the test–retest method (*r* = 0.8). In the present study, the same questionnaire structure, item content and 5‐point Likert response format were used, and no item‐level modification was made to the questionnaire.

Internal consistency reliability was calculated for the questionnaire version used in this study across all collected responses. Cronbach’s alpha was 0.95 for the total questionnaire and 0.84, 0.76 and 0.71 for the emotional support, physical support and informational/counselling support subscales, respectively.

The primary outcome was the total Observance of the Maternal Rights Charter in Labour score at baseline, 2 weeks and 2 months across each evaluator’s perspective. Secondary outcomes were the emotional, physical and informational/counselling support subscale scores across each evaluator’s perspective.

### 2.5. Intervention

Both groups continued to receive standard professional education and routine ward‐based educational activities ordinarily provided in the hospital. The intervention group received a structured, group‐based spiritual care empowerment programme.

The programme was developed through a structured and context‐sensitive process to ensure scientific rigour, cultural appropriateness and applicability to midwifery practice. The preliminary curriculum was derived from an educational booklet on spiritual care for healthcare providers approved by Qom University of Medical Sciences and further informed by the Iranian framework proposed by Heidari et al. [22] for developing a charter of spiritual care for patients. The draft programme was reviewed by a 10‐member multidisciplinary expert panel comprising specialists in reproductive health, medicine, social sciences, mental health nursing and psychiatric nursing, all of whom had research experience in spiritual health. Revisions were made based on expert feedback, and the final curriculum was approved after consensus was reached.

The intervention was delivered as an active, face‐to‐face workshop over 2 days. Two sessions were held each day, with each session lasting 2 h, for a total of 8 h. The workshop included PowerPoint‐based lectures, guided discussions, role‐playing, clinical experience sharing and question‐and‐answer activities. The four‐session workshop covered: (1) spirituality and spiritual health foundations, (2) principles and history of spiritual care, (3) communication in spiritual care and (4) practical competencies, including spiritual assessment and intervention. The full content is detailed in Table 1.

**TABLE 1:** Content of the spiritual care empowerment workshop.

Session	Content
First	Conceptual foundations of spirituality and spiritual health: epistemology and ontology of spirituality; the relationship between spiritual health and other dimensions of health; and a holistic view of health.
Second	Concepts, principles and background of spiritual care: concepts of spirituality, spiritual health and spiritual care; principles and key messages of spiritual care; and an overview of the history and development of spiritual care in Iran and internationally.
Third	Communication in spiritual care: the importance of communication in spiritual care; relational dimensions of human communication; and skills and strategies for effective spiritual communication in clinical settings.
Fourth	Practical competencies in spiritual care: importance and goals of spiritual history taking; methods and tools for spiritual history taking; factors and conditions affecting spiritual assessment; identification of spiritual needs; spiritual diagnosis; and appropriate spiritual interventions.

The workshop was delivered by two faculty members with academic and professional expertise in spiritual health and care. To reinforce learning, educational materials were shared with the intervention group through Eitaa and Telegram for 2 weeks after the workshop. The control group did not receive structured spiritual care education during the study period and continued routine ward‐based education only.

### 2.6. Data Collection Procedure

Data were collected at three time points in both groups: baseline, 2 weeks after the intervention and 2 months after the intervention. The control group was assessed at equivalent intervals. Assessments were conducted across morning, afternoon and night shifts using the same schedule and procedures in both groups.

Each midwife was assessed once at each time point, resulting in three assessments per midwife over the study period. At each assessment, one indexed labour episode was evaluated from four perspectives: the midwife herself, a peer assessor, the ward supervisor and the mother receiving care during the episode. Each assessment was based on a different labour episode involving a different mother.

Mother evaluations were completed by mothers whose labour care had been provided by the evaluated midwife. In most cases, the questionnaire was completed within the first 2 h after birth while the mother remained in the labour unit. When this was not possible, it was completed at the earliest feasible time within the first 24 h after birth while the mother was still hospitalised.

Peer assessments were completed by trained and experienced midwives from the same labour unit. Supervisor assessments were completed by the ward supervisor based on direct observations during the same indexed labour episode. Peer assessors and supervisors received standardised training on the questionnaire, scoring procedures and observation protocol before data collection.

Because of the educational nature of the intervention, participants and facilitators could not be blinded to group allocation, and outcome assessors could not be guaranteed to be blinded to group allocation. To reduce observer effects, assessments were conducted unobtrusively during routine care. Participating midwives were not informed of the exact timing of each assessment, and the evaluator forms were completed separately to preserve confidentiality and reduce the influence between raters. The four forms were analysed separately rather than being combined into a single composite score.

### 2.7. Ethical Considerations

This study was conducted in accordance with the principles of the Declaration of Helsinki (1964) and its amendments. The study protocol was approved by the Ethics Committee of Qom University of Medical Sciences (Qom, Iran; IR.MUQ.REC.1402.03). Written informed consent was obtained from all participants after they received a full explanation of the study objectives and procedures. Participants were assured that participation was voluntary, that they could withdraw from the study at any time without penalty and that their responses would be kept confidential and would not affect their employment status.

### 2.8. Data Analysis

Data were analysed using SPSS Version 26. Descriptive statistics, including the mean, standard deviation, frequency and percentage, were used to summarise the demographic and study variables. The normality of continuous variables was assessed before the inferential analysis. Independent samples *t*‐tests were used for continuous baseline variables, and chi‐square or Fisher’s exact tests were used for categorical variables as appropriate.

Group‐by‐time differences in Maternal Rights Charter scores were examined using a two‐way repeated‐measures analysis of variance, with group as the between‐subject factor and time as the within‐subject factor. The group‐by‐time interaction was used to determine whether changes over time differed between the intervention and control groups. Mauchly’s test indicated violations of the sphericity assumption in some repeated‐measures analyses; therefore, Greenhouse–Geisser‐corrected *p* values were reported where appropriate. Bonferroni‐adjusted pairwise comparisons were used for post hoc comparisons when appropriate. Partial eta squared (*η* *p* ^2^) was reported as the effect size.

The unit of assignment and statistical analysis was the midwife. Mother assessments were linked to indexed labour episodes and used as one of the four evaluator perspectives rather than as independent participant‐level units.

The final analysis was conducted as a complete‐case analysis. Participants with incomplete follow‐up data were excluded from the repeated‐measures analysis. In the final analysed dataset, no missing values were present for the computed outcome scores used in the repeated‐measures analyses. Statistical significance was set at *p* < 0.05.

## 3. Results

### 3.1. Demographic Characteristics

A total of 51 midwives were included in the final complete‐case analysis, with 23 in the intervention group and 28 in the control group. There were no statistically significant between‐group differences in the baseline demographic or occupational characteristics (Table 2).

**TABLE 2:** Baseline demographic and occupational characteristics of participating midwives.

Variable	Intervention group (*n* = 23)	Control group (*n* = 28)	Test statistic	*p* value
Continuous variables, mean (SD)				
Age, years	37.35 (6.02)	34.79 (5.50)	*t* = 1.59	0.119
Work experience, years	12.24 (5.11)	11.01 (6.02)	*t* = 0.74	0.460
Working hours per month	183.26 (23.62)	190.21 (16.49)	*t* = −1.23	0.223
Categorical variables, *n* (%)				
Marital status				
Single	3 (13.0)	10 (35.7)	*χ* ^2^ = 2.33	0.127
Married	20 (87.0)	18 (64.3)		
Education				
BA	21 (91.3)	21 (75.0)	*χ* ^2^ = 1.32	0.250
MA	2 (8.7)	7 (25.0)		
Income				
Below 10 million Toman	1 (4.3)	3 (10.7)	*χ* ^2^ = 1.06	0.588
10–15 million Toman	18 (78.3)	22 (78.6)		
Above 15 million Toman	4 (17.4)	3 (10.7)		
Working shift				
Morning	6 (26.1)	2 (7.1)	*χ* ^2^ = 5.46	0.065
Evening	0 (0.0)	3 (10.7)		
Rotating	17 (73.9)	23 (82.1)		
Employment				
Project based	0 (0.0)	2 (7.1)	*χ* ^2^ = 3.00	0.392
Contract based	3 (13.0)	4 (14.3)		
Company based	2 (8.7)	5 (17.9)		
Contractual/permanent	18 (78.3)	17 (60.7)		

*Note:* Values are presented as mean (SD) or *n* (%), as appropriate. Between‐group comparisons were performed using independent‐samples *t*‐tests for continuous variables and chi‐square or Fisher’s exact tests for categorical variables, as appropriate.

Abbreviations: BA, bachelor’s degree; MA, master’s degree.

### 3.2. Observance of the Maternal Rights Charter (360‐Degree Evaluation)

Tables 3 and 4 present the Maternal Rights Charter scores at baseline, 2 weeks and 2 months after the intervention based on self, peer, supervisor and mother evaluations, together with the repeated‐measures analysis of variance results. For the total scores, significant time‐by‐group interactions were observed across all four evaluator perspectives: self‐assessment (*F* = 22.38, *p* < 0.001, *η* *p* ^2^ = 0.314), peer assessment (*F* = 29.72, *p* < 0.00 1, *η* *p* ^2^ = 0.378), supervisor assessment (*F* = 41.74, *p* < 0.001, *η* *p* ^2^ = 0.460) and mother assessment (*F* = 21.56, *p* < 0.001, *η* *p* ^2^ = 0.306). From baseline to 2 months, total scores in the intervention group increased by 8.70 points in self‐assessment, 18.96 points in peer assessment, 19.66 points in supervisor assessment and 21.44 points in mothers’ assessments. The corresponding changes in the control group were −0.46, 0.61, −0.60 and 2.57, respectively. Significant time‐by‐group interactions were also observed for the emotional, informational/counselling and physical support dimensions across all four evaluator perspectives (Table 4).

**TABLE 3:** Maternal rights charter scores by group, time point and evaluator perspective.

Dimension/evaluator perspective	Intervention baseline	Intervention 2 weeks	Intervention 2 months	Control baseline	Control 2 weeks	Control 2 months
Emotional						
Self‐assessment	37.13 (5.47)	40.26 (4.92)	40.57 (4.01)	35.96 (7.04)	35.89 (6.84)	35.93 (6.95)
Peer	34.17 (4.14)	40.74 (7.21)	40.48 (7.42)	33.21 (5.12)	32.79 (4.91)	33.21 (5.12)
Supervisor	32.61 (2.39)	41.39 (5.13)	41.83 (6.75)	31.89 (4.47)	31.79 (3.59)	31.89 (4.47)
Mother assessment	30.43 (5.43)	37.09 (5.68)	37.57 (5.10)	29.86 (6.34)	30.46 (7.37)	30.00 (6.14)
Informational/counselling support						
Self‐assessment	33.96 (5.94)	36.87 (4.63)	37.26 (3.96)	33.57 (5.74)	33.25 (5.23)	33.14 (5.25)
Peer	32.91 (5.78)	39.30 (6.14)	39.48 (6.11)	32.36 (5.37)	32.61 (5.36)	32.96 (4.83)
Supervisor	33.65 (4.45)	38.87 (5.96)	38.87 (5.96)	32.36 (4.30)	32.00 (4.65)	32.00 (4.65)
Mother assessment	31.22 (7.38)	35.13 (5.12)	40.74 (4.69)	29.14 (8.00)	29.39 (7.98)	31.82 (6.06)
Physical						
Self‐assessment	24.70 (3.94)	26.96 (2.72)	26.65 (3.16)	24.43 (4.57)	24.57 (4.22)	24.43 (4.57)
Peer	24.52 (2.25)	31.43 (3.80)	30.61 (4.12)	23.07 (5.21)	23.57 (4.73)	23.07 (5.21)
Supervisor	24.04 (3.18)	29.70 (4.06)	29.26 (4.23)	22.89 (4.30)	23.36 (3.83)	22.64 (4.47)
Mother assessment	27.52 (3.94)	33.43 (2.09)	32.30 (2.91)	26.82 (4.77)	27.46 (4.08)	26.57 (4.48)
Total						
Self‐assessment	95.78 (13.25)	104.09 (10.89)	104.48 (9.60)	93.96 (16.10)	93.71 (14.84)	93.50 (15.21)
Peer	91.61 (9.52)	111.48 (14.83)	110.57 (15.76)	88.64 (13.33)	88.96 (12.46)	89.25 (12.98)
Supervisor	90.30 (8.34)	109.96 (13.51)	109.96 (15.88)	87.14 (10.09)	87.14 (9.21)	86.54 (10.89)
Mother assessment	89.17 (14.51)	105.65 (9.46)	110.61 (9.27)	85.82 (17.22)	87.32 (16.78)	88.39 (14.22)

*Note:* Values are presented as mean (SD).

**TABLE 4:** Repeated‐measures analysis of variance results for maternal rights charter scores.

Dimension/evaluator perspective	Group *F*	Group *p* value	Time *F*	Time *p* value	Time × group *F*	Time × group *p* value	*η* *p* ^2^
Emotional							
Self‐assessment	4.11	0.048	13.95	< 0.001	18.19	< 0.001	0.271
Peer	14.10	< 0.001	13.31	< 0.001	18.92	< 0.001	0.279
Supervisor	36.91	< 0.001	32.08	< 0.001	40.02	< 0.001	0.449
Mother assessment	10.25	0.002	16.41	< 0.001	15.96	< 0.001	0.245
Informational/counselling support							
Self‐assessment	3.85	0.056	5.58	0.015	11.82	< 0.001	0.194
Peer	11.30	0.002	15.03	< 0.001	13.60	< 0.001	0.217
Supervisor	14.92	< 0.001	14.42	< 0.001	23.85	< 0.001	0.327
Mother assessment	11.07	0.002	29.09	< 0.001	9.79	< 0.001	0.170
Physical							
Self‐assessment	2.35	0.132	6.98	0.008	7.10	0.008	0.127
Peer	24.12	< 0.001	36.21	< 0.001	36.07	< 0.001	0.424
Supervisor	21.09	< 0.001	23.27	< 0.001	24.99	< 0.001	0.342
Mother assessment	22.06	< 0.001	14.94	< 0.001	13.85	< 0.001	0.220
Total							
Self‐assessment	4.22	0.045	15.20	< 0.001	22.38	< 0.001	0.314
Peer	21.66	< 0.001	27.07	< 0.001	29.72	< 0.001	0.378
Supervisor	32.68	< 0.001	32.03	< 0.001	41.74	< 0.001	0.460
Mother assessment	16.65	< 0.001	29.09	< 0.001	21.56	< 0.001	0.306

*Note:* Two‐way repeated‐measures analysis of variance was used to test group, time and time‐by‐group interaction effects. Mauchly’s test was used to assess sphericity; Greenhouse–Geisser‐corrected *p* values are reported for within‐subject time effects and time‐by‐group interaction effects where appropriate. *η* *p* ^2^ = partial eta squared.

Mauchly’s test indicated a violation of the sphericity assumption for the repeated measures; therefore, Greenhouse–Geisser‐corrected *p* values are reported for the within‐subject time effects and time‐by‐group interaction effects in Table 4. The corrected analyses did not change the interpretation of the main findings of this study.

## 4. Discussion

This quasiexperimental study found that participation in the spiritual care empowerment programme was associated with improved midwives’ adherence scores for the Maternal Rights Charter across emotional, physical and informational/counselling dimensions. This pattern was observed across all four evaluator perspectives: self, peers, supervisors and mothers. Scores in the intervention group increased from baseline to 2 weeks and were maintained at 2 months, whereas scores in the control group showed only small changes over the same period. Significant time‐by‐group interactions across all evaluator perspectives, with moderate‐to‐large effect sizes, suggest that the observed changes may be practically meaningful.

These findings are important because adherence to the Maternal Rights Charter in maternity care is enacted through observable clinical behaviours rather than through formal awareness alone. Iranian evidence has examined the Maternal Rights Charter both as an educational/consultative focus for improving mothers’ perceptions of respectful maternity care and as a construct assessable through 360‐degree evaluation in labour and delivery settings [4, 12]. The observed improvement across the emotional, physical and informational/counselling dimensions is consistent with a possible strengthening of rights‐based maternity behaviours, including respectful communication, protection of privacy, provision of information, supportive responses to mothers’ needs and dignity‐preserving care. This interpretation is consistent with respectful maternity care frameworks, which conceptualise high‐quality childbirth care in terms of dignity, privacy, confidentiality, informed choice, support and freedom from harm and mistreatment, and operationalise these principles as observable provider‐level behaviours [2, 23].

The findings can also be interpreted in relation to selected evidence on spiritual care education and respectful maternity care training. Wang et al. [10] reported that nurses’ perceptions and competencies regarding spirituality and spiritual care were generally moderate, supporting the need for targeted theoretical and practical education in spiritual care. Dos Santos et al. [15] found that nurse‐delivered spiritual interventions in hospital and long‐term care settings often include components such as spiritual assessment, active listening, supportive presence and existential or religious support, although intervention content and outcome measurement varied substantially. In respectful maternity care, Dhakal et al. [24] concluded that educational interventions provide low‐level evidence for improving midwives’, nurses’ and students’ knowledge and attitudes, and reported quantitative evidence suggesting improvements in woman–provider communication; however, heterogeneity in intervention content, delivery, duration, timing and evaluation methods limited robust conclusions. The present study adds to this literature by examining adherence to the Maternal Rights Charter as a behavioural, 360‐degree‐assessed outcome in an Iranian maternity setting, rather than focussing primarily on knowledge, attitudes, perceptions or communication outcomes.

Based on the pattern of results, one plausible explanation is that the spiritual care empowerment programme may have increased midwives’ attentiveness to women as persons, including their dignity, values, beliefs, concerns, need for explanation, privacy and emotional distress. These elements overlap with spiritual care concepts such as spiritual assessment, active listening, supportive presence and responsiveness to patients’ beliefs and needs, and with provider‐level respectful maternity care behaviours such as respectful communication, information provision, privacy protection, consent and supportive care [2, 15, 25]. Evidence that nurses’ perceptions and competencies regarding spirituality and spiritual care are generally moderate supports the educational rationale for targeted spiritual care training [10]. However, the proposed explanation remains inferential because spiritual care competence, communication competence, relational attentiveness and other potential mediators were not measured directly in the present study.

The consistent pattern of improvement across evaluator groups is also noteworthy. Previous Iranian maternity care research using 360‐degree evaluation reported significant differences among self, peer, maternity officer/supervisor and parturient ratings of respect for the Bill of Mother’s Rights, with mothers and maternity officers reporting lower scores than midwives’ self‐ratings [12]. In the present study, improvements were observed from all four perspectives. This does not necessarily indicate complete agreement among raters; rather, it suggests that the programme‐associated changes were not limited to midwives’ self‐perceptions and were also detectable to peers, supervisors and mothers. This finding supports the value of multisource assessment for evaluating observable professional behaviour in ethically sensitive maternity care settings [12, 19].

For nursing and midwifery management, these findings suggest that spiritual care empowerment may be considered as one component of continuing professional development to support respectful, dignity‐centred and person‐centred maternity care. Training initiatives could move beyond general discussions of ethics or patients’ rights and include practical skills related to respectful communication, privacy‐preserving behaviours, responses to emotional and informational needs and ethically sensitive recognition of women’s spiritual concerns where relevant and acceptable to them. The use of 360‐degree evaluation may also help managers determine whether educational changes are perceived only by the staff or are also experienced by mothers receiving care [19].

This study had several strengths. Although participants were recruited by convenience sampling, eligible midwives were subsequently allocated to study groups using simple random allocation, which partially supported internal validity despite the single‐centre convenience sample. The 360‐degree evaluation framework, including self, peer, supervisor and mother assessments, reduced reliance on self‐reports and provided a broader assessment of observable professional behaviour. Repeated assessments at baseline, 2 weeks and 2 months after the intervention allowed for the evaluation of both immediate effects and effects maintained up to 2 months. Moreover, data collection during routine care and across different work shifts enhanced the clinical relevance of these findings.

This study has several limitations. It was conducted in a single maternity setting with a modest sample size, which may limit generalisability. The 2‐month follow‐up period prevents conclusions about the long‐term sustainability of the effects. Although the 360‐degree evaluation approach reduced reliance on self‐assessment alone, questionnaire‐based ratings may still have been influenced by response bias, social desirability or rater‐related factors. Mothers’ ratings may also have been affected by their overall childbirth experience, birth outcomes, ward conditions or aspects of care beyond individual midwives’ behaviour. Potential mediators, including spiritual care competence, communication competence, ethical sensitivity, previous spiritual care education, personal religiosity and ward ethical climate, were not measured directly. Therefore, the mechanisms underlying the observed improvements remain inferential. The absence of an active comparison group also limits conclusions about whether the effects were specific to spiritual care empowerment. Finally, attrition occurred only in the intervention group, which may have introduced selection bias and reduced statistical power.

In conclusion, participation in the spiritual care empowerment programme was associated with improved midwives’ adherence scores for the Maternal Rights Charter across dimensions and evaluator perspectives. The findings suggest that spiritual care empowerment may support relational attentiveness and may help make respectful, dignity‐centred and rights‐based behaviours more visible in routine maternity care. Because this was a single‐centre quasiexperimental study with complete‐case analysis and attrition only in the intervention group, the findings should be interpreted cautiously. Future multicentre studies with larger samples, longer follow‐ups, active comparison groups, intention‐to‐treat analyses and mediator analyses are recommended.

## Author Contributions

Zohre Khalajinia, Soheila Jafarzadeh and Hoda Ahmari Tehran conceived and designed the study. Zohre Khalajinia and Hoda Ahmari Tehran delivered the intervention. Zahra Alipour and Hoda Ahmari Tehran performed the data analyses. Zohre Khalajinia, Soheila Jafarzadeh and Zahra Alipour prepared the first draft of the manuscript. Zohre Khalajinia, Hoda Ahmari Tehran and Zahra Alipour critically revised the manuscript for important intellectual content.

## Funding

This study was supported by Qom University of Medical Sciences, Grant Number: IR.MUQ.REC.1402.03.

## Disclosure

All authors reviewed and approved the final manuscript. The funding body had no role in the design of the study, data collection, analysis, interpretation of the data or writing of the manuscript.

## Ethics Statement

This study was conducted in accordance with the principles of the Declaration of Helsinki. The study was approved by the Ethics Committee of Qom University of Medical Sciences (code: IR.MUQ.REC.1402.03).

## Consent

Written informed consent was obtained from all participants.

## Conflicts of Interest

The authors declare no conflicts of interest.

## Supporting Information

Additional supporting information can be found online in the Supporting Information section.

## Supporting information


**Supporting Information** Supplementary File 1. Completed TREND checklist for this single‐centre quasiexperimental study.

## Data Availability

The data supporting the findings of this study are available upon request from the corresponding author. The data are not publicly available because of privacy and ethical restrictions on data sharing.
