# Supplementary material for: Spiritual Care Empowerment and Midwives’ Adherence to the Maternal Rights Charter: A Quasiexperimental Study Using 360‐Degree Evaluation
Source: J Nurs Manag. 2026 Jul 24;2026:2125073. doi: 10.1155/jonm/2125073 (PMC13397467; doi:10.1155/jonm/2125073)
Supplement: Supplementary file 1 — Supporting Information Supplementary File 1. Completed TREND checklist for this single‐centre quasiexperimental study. [file JONM-2026-2125073-s001.docx]

Supplementary File 1. Completed TREND Statement Checklist

Manuscript: Spiritual Care Empowerment and Midwives' Adherence to the Maternal Rights Charter: A Quasi-Experimental Study Using 360-Degree Evaluation

| Paper section/topic | Item no. | TREND descriptor | Reported? | Page / line location | Notes for this manuscript |
| --- | --- | --- | --- | --- | --- |
| Title and Abstract | 1 | Information on how units were allocated to interventions; structured abstract recommended; information on target population or study sample. | Yes | Title, p. 1; Abstract, p. 2, lines 31-57. | The revised title identifies the quasi-experimental design and 360-degree evaluation. The structured abstract reports the midwife sample, allocation by lottery, final complete-case sample, intervention/control conditions, outcome assessment, and analysis. |
| Introduction - Background | 2 | Scientific background and explanation of rationale; theories used in designing behavioural interventions. | Yes | Introduction, pp. 3-5, lines 62-128. | The rationale is grounded in respectful maternity care, person-centred maternity care, health-system responsiveness, spiritual care competence, and 360-degree evaluation. |
| Methods - Participants | 3 | Eligibility criteria; method of recruitment; recruitment setting; settings and locations where data were collected. | Yes | Methods 2.1-2.2, pp. 5-6, lines 129-152; Data collection, pp. 7-8, lines 209-233. | Eligibility criteria, exclusion criteria, convenience/consecutive recruitment of midwives, labour and delivery ward setting, and data collection across shifts are reported. |
| Methods - Interventions | 4 | Details of each study condition, including content, delivery method, unit of delivery, deliverer, setting, exposure quantity and duration, time span, and adherence/compliance activities. | Yes | Methods 2.5, p. 7, lines 184-208; Table 1, p. 17, line 521. | The intervention is described as a group-based, face-to-face 8-hour workshop over two days, delivered by two faculty members, with two weeks of educational support through Eitaa and Telegram. The control group received routine ward-based education only. |
| Methods - Objectives | 5 | Specific objectives and hypotheses. | Yes | Abstract, p. 2, lines 31-33; Introduction, p. 5, lines 121-128. | The objective was revised to examine whether participation in the program was associated with changes in adherence to the Maternal Rights Charter over time. |
| Methods - Outcomes | 6 | Clearly defined primary and secondary outcome measures; methods used to collect data and enhance measurement quality; information on validated instruments and psychometric properties. | Yes | Methods 2.4, pp. 6-7, lines 162-183; Data collection, pp. 7-8, lines 209-226. | Primary and secondary outcomes are explicitly defined. The 34-item questionnaire, dimensions, response format, validity source, test-retest reliability, and internal consistency in the present study are reported. |
| Methods - Sample Size | 7 | How sample size was determined and, when applicable, explanation of interim analyses and stopping rules. | Yes | Methods 2.3, p. 6, lines 153-161. | The a priori sample size calculation, assumptions, attrition allowance, and final recruitment target are described. Interim analyses/stopping rules were not applicable. |
| Methods - Assignment Method | 8 | Unit of assignment; method used to assign units to study conditions; restrictions and bias-minimisation aspects if used. | Yes | Methods 2.2, pp. 5-6, lines 144-152; Data analysis, p. 9, lines 256-258. | The unit of assignment was the midwife. Participants were assigned 1:1 by simple random allocation using lottery. The analysis unit is also specified as the midwife. |
| Methods - Blinding/Masking | 9 | Whether participants, those administering interventions, and outcome assessors were blinded; if so, how blinding was accomplished and assessed. | Yes | Data collection, p. 8, lines 227-233. | The manuscript states that blinding was not possible/guaranteed because of the educational nature of the intervention, and describes measures to reduce observer effects. |
| Methods - Unit of Analysis | 10 | Smallest unit analysed; if the unit of analysis differs from the unit of assignment, analytical method used to account for this. | Yes | Data analysis, p. 9, lines 256-258. | The midwife was the unit of assignment and statistical analysis. Mother assessments were treated as evaluator perspectives linked to indexed labour episodes, not independent participant-level units. |
| Methods - Statistical Methods | 11 | Statistical methods for group comparisons and correlated data; additional analyses; imputation methods; statistical software. | Yes | Data analysis, p. 9, lines 242-262. | SPSS version 26, descriptive statistics, baseline t-tests/chi-square/Fisher tests, two-way repeated-measures ANOVA, Greenhouse-Geisser correction, Bonferroni adjustment, ηp² effect size, complete-case analysis, and missing-data handling are reported. No imputation or subgroup analyses were performed. |
| Results - Participant Flow | 12 | Flow through enrolment, assignment, allocation/intervention exposure, follow-up, analysis; protocol deviations with reasons; diagram strongly recommended. | Yes | Methods 2.2, pp. 5-6, lines 144-152; Results, p. 9, lines 264-267; Figure 1, p. 19, lines 532-534. | The text and Figure 1 report enrolment, allocation, intervention/control exposure, attrition with reasons, follow-up, and final complete-case analysis. |
| Results - Recruitment | 13 | Dates defining the periods of recruitment and follow-up. | Yes | Abstract, p. 2, lines 38-39; Methods 2.1, p. 5, lines 131-135. | The study period is reported as May to August 2023. |
| Results - Baseline Data | 14 | Baseline demographic and clinical characteristics for each condition; baseline comparisons of those lost to follow-up and retained; comparison of study population with target population. | Yes / partly | Results 3.1, p. 9, lines 264-267; Table 2, p. 17, lines 522-525; Limitations, pp. 12-13, lines 362-374. | Baseline demographic and occupational characteristics are reported by condition and compared statistically. Attrition is described and discussed as a limitation; separate baseline comparisons of retained versus excluded participants were not reported because attrition was small and occurred only in the intervention group. |
| Results - Baseline Equivalence | 15 | Data on study group equivalence at baseline and statistical methods used to control for baseline differences. | Yes | Results 3.1, p. 9, lines 264-267; Table 2, p. 17, lines 522-525. | No statistically significant between-group differences in baseline demographic or occupational characteristics were reported. |
| Results - Numbers Analysed | 16 | Number of participants included in each analysis for each condition; indication of intention-to-treat or alternative strategy and how non-compliers were treated. | Yes | Methods 2.2, pp. 5-6, lines 144-152; Data analysis, p. 9, lines 259-262; Results, p. 9, lines 264-267; Figure 1, p. 19, lines 532-534. | The final complete-case sample was 51 midwives: 23 intervention and 28 control. Participants with incomplete follow-up data were excluded from repeated-measures analyses. |
| Results - Outcomes and Estimation | 17 | For primary and secondary outcomes, summary results for each condition, estimated effect size and precision; inclusion of null/negative findings; causal pathway testing if any. | Yes | Results 3.2, pp. 9-10, lines 268-280; Table 3, pp. 17-18, lines 526-527; Table 4, p. 18, lines 528-531; Discussion, p. 11, lines 322-333. | Means/SDs by group and time are reported, with F statistics, p-values, and ηp² effect sizes for group, time, and time-by-group effects. Corrected mother-assessment total scores and ANOVA results are reported. Potential mechanisms are discussed as theoretical rather than directly tested. |
| Results - Ancillary Analyses | 18 | Summary of other analyses, including subgroup or restricted analyses, indicating which were prespecified or exploratory. | N/A | No ancillary/subgroup analyses were conducted. | The manuscript reports the prespecified complete-case repeated-measures analyses only. |
| Results - Adverse Events | 19 | Summary of important adverse events or unintended effects in each condition. | N/A | Not applicable to this educational workshop study. | The study evaluated an educational professional-development intervention; no clinical intervention or adverse-event outcome was planned. |
| Discussion - Interpretation | 20 | Interpretation of results considering hypotheses, potential bias, imprecision, multiple analyses, limitations, mechanisms/alternative explanations, implementation, and implications. | Yes | Discussion, pp. 10-13, lines 285-382. | The Discussion interprets findings cautiously, discusses possible mechanisms, acknowledges single-centre design, complete-case analysis, attrition only in the intervention group, lack of active comparator, unmeasured mediators, and implications for nursing/midwifery management. |
| Discussion - Generalizability | 21 | External validity considering study population, intervention characteristics, follow-up, site/setting, compliance, and contextual issues. | Yes | Limitations and conclusion, pp. 12-13, lines 362-382. | Generalisability limitations are discussed in relation to the single maternity setting, modest sample size, and two-month follow-up. |
| Discussion - Overall Evidence | 22 | General interpretation in the context of current evidence and theory. | Yes | Discussion, pp. 10-12, lines 294-352; Conclusion, p. 13, lines 375-382. | The results are interpreted in relation to respectful maternity care frameworks, spiritual care education evidence, multisource feedback theory, and implications for continuing professional development. |

*Source of checklist items: TREND Statement checklist, adapted from Des Jarlais et al. (2004), Am J Public Health, 94, 361-366.*
